# Choroid plexus-selective inactivation of adenosine A_2A_ receptors protects against T cell infiltration and experimental autoimmune encephalomyelitis

**DOI:** 10.1186/s12974-022-02415-z

**Published:** 2022-02-18

**Authors:** Wu Zheng, Yijia Feng, Zhenhai Zeng, Mengqian Ye, Mengru Wang, Xin Liu, Ping Tang, Huiping Shang, Xiaoting Sun, Xiangxiang Lin, Muran Wang, Zhengzheng Li, Yiyun Weng, Wei Guo, Sergii Vakal, Jiang-fan Chen

**Affiliations:** 1 Molecular Neuropharmacology Laboratory, School of Optometry and Ophthalmology and Eye Hospital, Wenzhou Medical University, Wenzhou, 325000 China; 2 Department of Neurology, The First Affiliated Hospital of Wenzhou Medical University, Wenzhou, 325000 Zhejiang China; 3 State Key Laboratory of Optometry and Vision Science, Wenzhou, 325000 China

**Keywords:** Adenosine A_2A_ receptor, Multiple sclerosis, Choroid plexus, Immune infiltration

## Abstract

**Background:**

Multiple sclerosis (MS) is one of the most common autoimmune disorders characterized by the infiltration of immune cells into the brain and demyelination. The unwanted immunosuppressive side effect of therapeutically successful natalizumab led us to focus on the choroid plexus (CP), a key site for the first wave of immune cell infiltration in experimental autoimmune encephalomyelitis (EAE), for the control of immune cells trafficking. Adenosine A_2A_ receptor (A_2A_R) is emerging as a potential pharmacological target to control EAE pathogenesis. However, the cellular basis for the A_2A_R-mediated protection remains undetermined.

**Methods:**

In the EAE model, we assessed A_2A_R expression and leukocyte trafficking determinants in the CP by immunohistochemistry and qPCR analyses. We determined the effect of the A_2A_R antagonist KW6002 treatment at days 8–12 or 8–14 post-immunization on T cell infiltration across the CP and EAE pathology. We determined the critical role of the CP-A_2A_R on T cell infiltration and EAE pathology by focal knock-down of CP-A_2A_R via intracerebroventricular injection of CRE-TAT recombinase into the A_2A_R^flox/flox^ mice. In the cultured CP epithelium, we also evaluated the effect of overexpression of A_2A_Rs or the A_2A_R agonist CGS21680 treatment on the CP permeability and lymphocytes migration.

**Results:**

We found the specific upregulation of A_2A_R in the CP associated with enhanced CP gateway activity peaked at day 12 post-immunization in EAE mice. Furthermore, the KW6002 treatment at days 8–12 or 8–14 post-immunization reduced T cell trafficking across the CP and attenuated EAE pathology. Importantly, focal CP-A_2A_R knock-down attenuated the pathogenic infiltration of Th17^+^ cells across the CP via inhibiting the CCR6–CCL20 axis through NFκB/STAT3 pathway and protected against EAE pathology. Lastly, activation of A_2A_R in the cultured epithelium by A_2A_R overexpression or CGS21680 treatment increased the permeability of the CP epithelium and facilitated lymphocytes migration.

**Conclusion:**

These findings define the CP niche as one of the primary sites of A_2A_R action, whereby A_2A_R antagonists confer protection against EAE pathology. Thus, pharmacological targeting of the CP-A_2A_R represents a novel therapeutic strategy for MS by controlling immune cell trafficking across CP.

**Supplementary Information:**

The online version contains supplementary material available at 10.1186/s12974-022-02415-z.

## Introduction

Multiple sclerosis (MS) is one of the most common autoimmune disorders characterized by the over-activated immune system with immune infiltration, demyelination and subsequent defects in cognition, vision, motor and sensory sensitivity [1]. Experimental autoimmune encephalomyelitis (EAE) resembles the over-activated immune system in MS pathology with specific CD4^+^ T helper cells response to myelin oligodendrocyte glycoprotein (MOG) antigen, and T cell infiltration, neuroinflammation, demyelination, and hindlimb paralysis. The recognition of MS/EAE as an inflammatory disease, characterized by infiltration of immune cells into the brain has spurred an investigation into the development of immunomodulatory treatment and led to the approval of three classes of immunomodulatory drugs for the treatment of MS, namely, (1) drugs depleting or impairing immune cells, (2) drugs modifying the activity of immune cells, and (3) drugs impairing the trafficking ability of lymphocytes into the brain [2, 3]. Among them, natalizumab, an antibody directed against VLA-4, blocks leukocyte infiltration into the brain across the blood–brain barrier (BBB) and reduces the annualized MS relapse rate by about 70% [4]. The therapeutic success of natalizumab constitutes the best proof-of-principle for leukocyte trafficking blockade as a valid approach to treat neuroinflammatory diseases. However, this treatment is associated with an unanticipated life-threatening adverse effect (progressive multifocal leukoencephalopathy) and requires a strict monitoring program. Thus, a search for alternative targets/strategies to control trans-epithelium/endothelium trafficking of immune cells into the brain is critically needed to develop an effective treatment of MS with a better safety profile.

Immune cells infiltrate over cellular barriers into the CNS (central nervous system) parenchyma through the BBB and choroid plexus (CP). While many autoimmune studies have mainly focused on the transmigration of T lymphocytes through the blood–brain barrier, the choroid plexus is the primary site of the entry for EAE-inducing Th17 cells into the brain [5] and may act as a hub for the regulation of CNS immune homeostasis in MS pathology [5, 6]. The monolayer and continuous line of the CP epithelium constitutes a tight junction (TJ) barrier preventing the entry of large molecules and maintaining the intracerebral homeostasis [7]. This CP epithelium lies in a connective and highly vascularized stroma populated by diverse cell types (fibroblasts, macrophages and dendritic cells). Increasing evidence supports that the choroid plexus is an essential route for the entry of immune cells. In MS/SPMS patients and optic neuritis patients, the cerebrospinal fluid (CSF) contains higher numbers of immune cells [8]; moreover such patients also display immune activation of the CP with mature immune cells in the CSF as evidenced by the increased expression of HLA-DR, CD86, CD80, and CD40 [9]. In the EAE model, immunization with myelin oligodendrocyte glycoprotein triggers the rapid accumulation of CD11C^+^ immune cells in the CP, prior to the onset of EAE clinical signs [9]. The immune cell infiltration across the CP is closely associated with altered expression of lymphocytes trafficking determinants in the CP [7, 10, 11]. Thus, the CP is a critical entry point for immune cells in MS, and targeting the CP gateway activity may be an effective MS treatment strategy.

This brings the adenosine A_2A_ receptor (A_2A_R), a potent regulator of neuroinflammation, in the CP into the focus of new therapeutic development for MS by controlling T cell infiltration. The study using positron emission tomography (PET) imaging with a radioligand to A_2A_R showed that A_2A_Rs were increased in the brain of secondary progressive multiple sclerosis (SPMS) patients [12]. The direct evidence for A_2A_R involvement in MS immunopathogenesis comes from our studies with EAE animals. We showed that the A_2A_R selective antagonist (SCH58261) and non-selective antagonist (caffeine) consistently attenuated EAE pathological process, in direct contrast to the A_2A_R-KO (knockout) observations [13–15]. However, genetic deletion of A_2A_Rs exacerbates the severity of EAE, with greater motor paralysis, more infiltrating CD4^+^ T lymphocytes in the CNS, and more severe demyelination [16]. We further investigated this contradiction via a series of adoptive transfer experiments using the radiation bone marrow chimera model system in which A_2A_R activity in T lymphocytes promoted the severity of the inflammatory response in EAE, while A_2A_R activity in radiation-resistant, non-hematopoietic cells limited the severe EAE pathology [17]. This suggests that exacerbation of EAE pathology by genetic deletion of A_2A_R can be largely attributed to A_2A_R action in lymphocytes with the promotion of inflammatory responses while attenuation of EAE pathology by pharmacological A_2A_Rs blockade owns to A_2A_R action on non-hematopoietic cells. Critically, we noted that the choroid plexus epithelium (CPE) expresses a high level of mRNAs for A_2A_R and CD73 (a 5′-nucleotidase converting AMP to extracellular adenosine and required for lymphocyte trafficking into the CNS), as revealed by fluorescence in situ hybridization (FISH) [17, 18]. The NECA (non-specific adenosine receptor agonist) regulates the expression of *cx3cl1,* which is causally linked to the protection against EAE induction as revealed by the CX3CL10 blocking antibody [19]. Thus, we proposed that the CP may represent a non-hematopoietic site where A_2A_R antagonists control T cell infiltration and EAE pathology.

In this study, we found that A_2A_R signal was upregulated in the CP tissues at day 8–12 post-immunization. In agreement with this time window, the selective treatment with the A_2A_R antagonist KW6002 at post-immunization day 8–12 or 8–14 was sufficient to reduce T cell trafficking into the CNS across the CP and attenuate the brain damage. Importantly, selective depletion of A_2A_R in the CP (by ICV-injection of CRE-TAT recombinant protein into A_2A_R^flox/flox^ mice) reduced the T cell trafficking into the CNS and alleviated EAE pathology by inhibiting the CCR6–CCL20 axis through NF-κB/STAT3 pathway. This CP-A_2A_R effect was at least partially mediated by direct A_2A_R action on the CP epithelium since boosted A_2A_R signaling in the CP epithelium increased its permeability in vitro and facilitated T cell migration in vivo. Collectively, these findings define the CP niche as one of the primary sites for A_2A_R action, whereby A_2A_R antagonist confers protection against EAE pathology. Thus, pharmacological targeting of the CP A_2A_R represents a novel therapeutic strategy for MS treatment by controlling immune cell trafficking across the CP.

## Materials and methods

### Animals

Eight-week-old C57BL/6 mice (female) were purchased from Charles River Laboratories (CRL, Beijing, China). The transgenic line B6.Cg-Gt (ROSA) 26Sor^tm9 (CAG−tdTomato) Hze^/J (Ai9) was purchased from Jackson Laboratory (Stock No: 007909). A_2A_R^flox/flox^ mice (female) have been described previously [20]. All mice were bred and housed under pathogen-free conditions at Wenzhou Medical University.

### EAE induction and behavioral scoring

The EAE model was produced following the procedure described previously [16]. Briefly, 1:1 emulsion of MOG_35–55_ peptide (1 mg/ml in PBS) (Anaspec) and complete Freund’s adjuvant (CFA, Sigma) was injected subcutaneously (50 μl) into each flank. In addition, pertussis toxin (PTX, 20 ng) (Biological Laboratories Inc.) was given intravenously (200 μl in PBS) at the time of immunization and again 2 days later. The EAE mice were scored daily based on the disease symptom severity scale (from day 0 to day 20 or 25), as we described previously [21].

### In vivo assay of the CP permeability

Briefly, at day 8, 10, or 12 post-immunization, the EAE mice were anesthetized with 4.5% isoflurane and then treated with intravenous injection (retro-orbitally) of 100 µl of 4 kDa Dextran Alexa Fluor 488 (final concentration 2 mg/100 µl). Fifteen minutes later, the mice were deeply anesthetized with isoflurane and then perfused with cold PBS and 4% formaldehyde (PFA). The brains were dissected out and embedded with O.C.T. matrix. The frozen section (20 µm thick) were used to evaluate the fluorescence intensity with a confocal microscope (LSM880, Zeiss).

### A_2A_R antagonist treatment

For the treatment with the A_2A_R antagonist KW6002 in vivo, mice were intraperitoneally injected with vehicle (DMSO/PBS) or KW6002 (5 mg/kg) every day. To isolate the effective time window of KW6002 treatment, mice were intraperitoneally injected with vehicle (DMSO/PBS) or KW6002 (5 mg/kg) every day either in the phase of day 8–14 or 8–12 post-immunization.

### Over-expression of A_2A_R in the CP cell line ZM310

The A_2A_R gene *Adora2a* (mouse, NM_009630.3) was cloned into the vector FV115 (CMV-MCS-3Flag-Ubi-ZsGreen-IRES-Puromycin) with the seamless cloning method and was used for the lentivirus package (1.1 × 10^8^). The empty vector was used as the control group.

The Z310 cells (2 × 10^4^) were cultured in a DMEM medium (DMEM/F12 and 10% fetal bovine serum) at 37 °C for 24 h. The cells were then transfected with the constructed lentivirus (MOI (multiplicity of infection) = 20). Forty-eight hours following the transfection, the A_2A_R-positive cells were screened with various concentrations of puromycin (1 μg/ml, 2.5 μg/ml, 5 μg/ml, 10 μg/ml). The ZsGreen-positive cells were then used for TEER and cell migration assays.

### Primary culture of the CP epithelium

The CP cell cultures were produced as described previously [22]. Briefly, following perfusion with PBS, the CP tissues were dissected and digested with 0.25% trypsin. The cells were cultured in Dulbecco’s modified Eagle’s medium (DMEM)/Ham’s F12 medium (Invitrogen) containing 10% fetal bovine serum (FBS), 1 mM l-glutamine, 1 mM sodium pyruvate, penicillin/streptomycin (100 U/ml), insulin (5 ng/ml), 20 μM arabinosylcytosine (Ara-C), sodium selenite (5 ng/ml), and epidermal growth factor (EGF) (10 ng/ml) (Sigma-Aldrich). Then, the cells were cultured in the L-polylysine-coated 24-well plates or polycarbonate filters according to the experimental goal.

### Effects of CGS21680 and NF-kB/STAT3 inhibitor

Following cultivation of the above-mentioned primary CP cells for three days, the cells were treated with CGS21680 (100 nM), or vehicle (DMSO). Then, 2 h later, the cells were collected for qPCR or Western Blot. In addition, the 3-day-cultured primary CP cells were pretreated with JSH-23 (10 μM, a NFκB transcriptional activity inhibitor, 749886-87-1, Sigma-Aldrich), Stattic (100 μM, a specific STAT3 inhibitor, 573099, Millipore) or vehicle for 1 h. Then, CGS21680 (100 nM, C141, Sigma-Aldrich) or vehicle was added. Finally, 2 h later, the cells were collected for qPCR.

### TEER measurement

The primary CP cells were isolated as described above and cultured on polycarbonate filters in Transwell chamber (pore size: 5 μm, diameter: 6.5 mm). Six days later, the A_2A_R agonist CGS21680 (100 nM) was added into the Transwell. Then, TEER was monitored within 2 h (MERS00002, Millipore). For the rat CP epithelium cell line (ZM310), the A_2A_R-labeled, empty vector-labeled or blank ZM310 cells were cultured on the same filters for 4 days, then TEER was measured.

### T cell migration assay

The method was used as described previously [23]. Briefly, the A_2A_R-labeled, empty vector-labeled or blank ZM310 cells were grown on filters as mentioned above for 3 days. The CD4^+^ T cells were isolated from the spleen with Anti-Mouse CD4 Magnetic Particles (BD, #551539). Then, the medium of the upper chamber was changed to 100 μl 1640 medium containing CD4^+^ T cells (1 × 10^6^ cells/ml), and the insert was plated into a new well filled with RPMI 1640 (0.5% FBS). Twenty-four hours later, the migrating cells in the lower chamber were subjected to crystal violet staining and counted under a microscope (DM750, Leica).

### Quantitative real-time PCR (qPCR)

Following perfusion with PBS, the CP was dissected from the ventricles for RNA isolation using Trizol (Invitrogen) and cDNA synthesis with PrimeScript™ 1st Strand cDNA synthesis Kit (Takara, 6110A). Quantitative PCR was performed with SYBR-Green premix Extaq (Takara) and detected with a Real-Time PCR System (CFX96; Bio-Rad). The following primers were used: *ppia* (forward 5′-AGCATACAGGTCCTGGCATCTTGT-3′ and reverse 5′ -CAAAGACCACATGCTTGCCATCCA-3′), *icam1* (forward 5′-AGATCACATTCACGGTGCTGGCTA-3′ and reverse 5′-AGCTTTGGGATGGTAGCTGGAAGA-3′), *ccl2* (forward 5′-CATCCACGTGTTGGCTCA-3′ and reverse 5′-GATCATCTTGCTGGTGAATGAGT-3′), *cxcl10* (forward 5′-AACTGCATCCATATCGATGAC-3′ and reverse 5′-GTGGCAATGATCTCAACAC-3′), *ccl5* (forward 5′-GCTGCTTTGCCTACCTCTCC-3′ and reverse 5′-TCGAGTGACAAACACGACTGC-3′), *ccl20* (forward 5′- GCCTCTCGTACATACAGACGC-3′ and reverse 5′- CCAGTTCTGCTTTGGATCAGC-3′), and *adora2a* (forward, 5′-CCGAATTCCACTCCGGTACA-3′ and reverse 5′-CAGTTGTTCCAGCCCAGCAT-3′). The C_t_ values were converted to relative quantification data using a 2^−ΔΔCt^ method.

### Immunofluorescent and hematoxylin–eosin (H&E) staining

Anesthetized mice were fully perfused with PBS and 4% PFA (paraformaldehyde) and the brains were isolated and fixed with PFA. Before staining, the brain slices (10 μm thick) were washed with PBS three times and then incubated for 30 min in PBS containing 0.3% Triton X-100 and 3% serum. Later, the slices were incubated with primary antibodies for 12 h: rabbit anti-ICAM1 (1:100, Abcam) or mouse anti-A_2A_R (1:300, Wako). The sections were then washed with PBS and incubated for 2 h at room temperature with Alexa Fluor 488 antibodies (Abcam; 1:400). The slices were then washed and mounted on slides with VECTASHIELD mounting media (containing DAPI). For CD3 staining, the isolated CP tissues were fixed with 2.5% PFA for 30 min and then transferred to PBS. The following protocol was similar to the above procedure with rat anti-CD3 (1:100, CD3-FITC, Abcam). Images were acquired with a confocal microscope (LSM880, Zeiss). As described earlier [16], tissue slices were stained with hematoxylin–eosin.

### CP-specific knock-down of A_2A_Rs

The focal knock-down of A_2A_Rs in the CP was achieved using the method described previously [24]. In brief, 2.11 μl of CRE-TAT recombinase (20 μg for each ventricle, Millipore) or sterile PBS were injected into each of the lateral ventricles (AP: 0.98, ML: − 1.3, DV: 2.6). The injection rate was 1 μl/min, and the needle was kept inside the brain for other 5 min before a withdrawal. Mice were allowed to recover for 2 weeks before the MOG immunization.

### Flow cytometry analysis of immune cells

Following perfusion with PBS, the spleen, CSF (5 μl per mouse) and spinal cord were isolated from PBS or CRE-TAT treated mice at day 14 post-immunization. Cell suspensions of the spleen and spinal cord were prepared and incubated with ACK buffer (C3702, Beyotime) to lyse red blood cells. With a centrifugation at 1500 rpm for 5 min, the cells were resuspended with 100 μl of staining buffer (70-S1001, Multisciences). Next, 10 μl of Clear Back (FcR blocking, MTG-001, MBL) were mixed with 50 μl of cell suspension (≤ 1 × 10^6^) and incubated for 5 min at room temperature. Then, the samples were stained with fluorochrome-conjugated monoclonal antibodies against FVS510 (564406, BD Pharmingen), CD3-FITC (11-0032-82, Invitrogen), CD4-PerCP-Cy5.5 (550954, BD Pharmingen) and CD196-AF647 (561753, BD Pharmingen) for 30 min at 4 °C. Next, the staining buffer (3 ml per sample) was added into the sample solution and mixed well. Following a centrifugation at 1500 rpm for 5 min, the cells were resuspended with 200 μl of staining buffer. At last, the stained cells were acquired with a flow cytometer (NovoCyte Quanteon, Agilent) and analyzed with NovoExpress software (Agilent).

### Western blot analysis of signaling molecules

The primary CP cells were isolated as stated above and cultured in L-polylysine-coated plates for 2 days. Then, the cells were, respectively, treated with vehicle, 100 nM CGS21680 (HY-13201, MCE), 30 μM JSH-23 (an inhibitor of nuclear translocation of p65) (HY-13982, MCE), 20 μM Stattic [an inhibitor of STAT3 phosphorylation (Y705 and S727)] (HY-13818, MCE), a combination of JSH-23 (30 μM) and CGS21680 (100 nM), or a combination of Stattic (20 μM) and CGS21680 (100 nM). The cells were collected and prepared for RNA or protein isolation. The proteins were transferred from polyacrylamide gels to PVDF membranes (Invitrogen) at 100 V for 120 min. Membranes were blocked using bovine serum albumin (5% w/v). Membranes were then incubated with primary antibodies overnight at 4 °C and secondary antibodies at room temperature for 90 min. The following primary antibodies were used: anti-Stat3 (12640S, CST; 1:1000, rabbit), anti-Phospho-Stat3 (Tyr 705) (9145S, CST; 1:1000, rabbit), anti-NFκB-p65 (8242S, CST; 1:1000, rabbit) and anti-Phospho-NFκB-p65 (Ser 536) (3033S, CST; 1:1000, rabbit) (Ser 536) (9145S, CST; 1:1000, rabbit). The secondary antibody was IRDyer 800CW (LICOR; 1:5000). Signal intensities were calculated using ImageJ software and normalized to the values of Stat3 or p65.

### Statistical analyses

Statistical analysis of behavioral deficit scores in different groups during the EAE disease courses was performed by utilizing two-way ANOVA for repeated measurements (RM). One-way ANOVA was used to estimate leukocyte trafficking determinant expression during the EAE disease course. The Student’s t-test was used to compare two groups of drug treatment, over-expression or CRE-TAT injection. All statistical analyses were performed with GraphPad Prism 6.0.

## Results

### The concordance of elevated A_2A_R signal and enhanced T cell infiltration in the CP during EAE

**Fig. 1:**
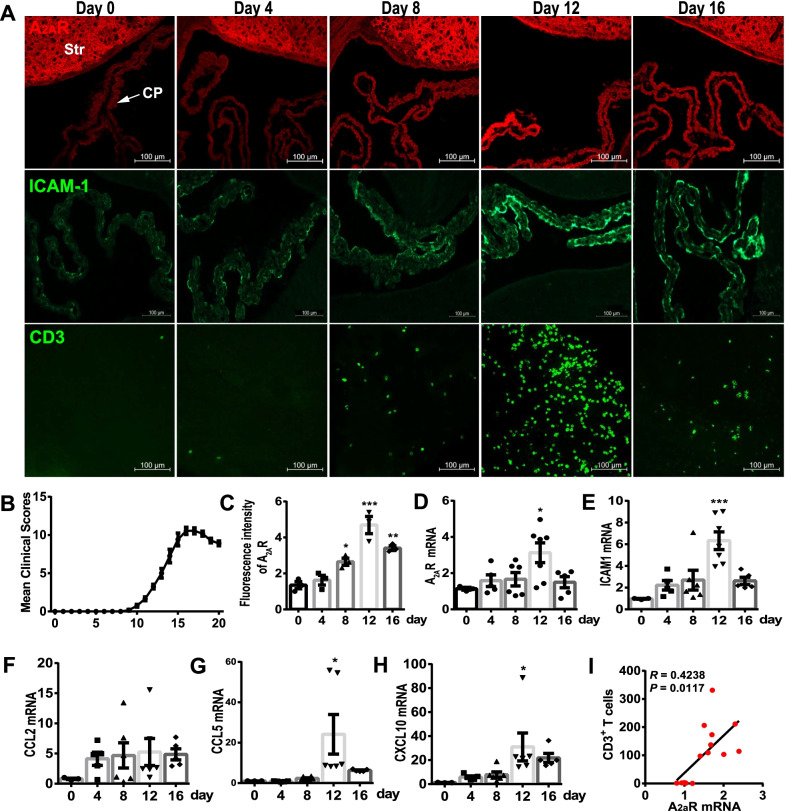
A_2A_R signals located on the choroid plexus were increased in the EAE model. **A** Following EAE induction in WT mice, the immunofluorescent staining of A_2A_R, ICAM1 and CD3 was performed at day 0, 4, 8, 12, and 16 after immunization. Scale bar, 100 µm (*n* = 3/group). **B** The clinical scores of EAE mice in **A** (*n* = 20). **C** The fluorescence quantification of A_2A_R in **A** (*n* = 3/group). One-way ANOVA, Tukey’s multiple comparisons test. **D–H** The mRNA analysis of *adora2a* (A_2A_R), *icam1*, *ccl2*, *ccl5* and *cxcl10* at day 0 (*n* = 3), 4 (*n* = 5), 8 (*n* = 6), 12 (*n* = 7), and 16 (*n* = 5) post-immunization. **I** Regression analysis between the numbers of CD3^+^ T cells recruited to the CP tissue and mRNA level of *adora2a* (A_2A_R) (*n* = 14). *p < 0.05, **p < 0.01 and ***p < 0.001

As the CP is considered as the key site for the first wave of T cell trafficking into the brain [5], we firstly studied the expression of A_2A_R at different post-immunization time-points: day 0, 4, 8 (EAE pre-onset), 12 (ascent stage of pathology) and 16 (the peak season) by immunofluorescence staining (Fig. 1A, B). Interestingly, we found the low basal expression of A_2A_R (day 0) in the CP (Fig. 1A), comparable to that in the hippocampus and cortex [17], in contrast to the high expression level of A_2A_R in the striatum (Additional file 1: Fig. S1) [25]. A_2A_R expression was visibly elevated in 8–16 days and peaked at day 12 (Fig. 1A and C). In parallel with the CP-A_2A_R expression, ICAM-1 (intercellular cell adhesion molecule-1), a ligand of LFA-1 (lymphocyte function-associated antigen-1), displayed similar expression patterns, with the peak expression at post-immunization day 12, suggesting the enhanced immune adhesion for leukocytes migration (Fig. 1A). Moreover, we studied the events of T lymphocytes trafficking into the CNS across the CP by CD3 antibody. We found that CD3^+^ T cells dramatically aggregated in the CP at day 12 and the amount of CD3^+^ T cells decreased at day 16 (Fig. 1A). There was also a detectable increase in the number of T cells in the CP at day 8 when the mice displayed no pathological scores (Fig. 1A).

Notably, T cell infiltration into the CNS through the CP probably happened earlier than through the BBB. H&E staining revealed leukocyte infiltration in the CP as early as post-immunization day 8 with a peak at post-immunization day 12 following EAE induction (Additional file 2: Fig. S2A). By contrast, the “perivascular cuffs’’ (i.e., leukocyte cluster surrounding parenchyma vasculature) did not appear in the brain parenchyma until day 12 (Additional file 2: Fig. S2A). This early onset of CP/BCSFB (blood–CSF barrier) leakage compared to BBB was confirmed by dextran. After EAE induction, clear FITC signals were detected in the CP at day 8 and increased at days 10 and 12 (Additional file 2: Fig. S2B). However, the FITC signals were nearly undetectable around the BBB in the brain parenchyma at day 8, but gradually appeared at days 10 and 12 (Additional file 2: Fig. S2B).

The qPCR analysis further suggested that A_2A_R signals, as well as chemotaxis and adhesion molecules (*icam1*, *ccl5* and *cxcl10*) were upregulated at days 8–16 (Fig. 1D–H). Following EAE induction, we isolated the PBS-perfused CPs at day 12. For each mouse, we determined the number of CD3^+^ T cells in the CP from one hemisphere, and the A_2A_R mRNA level in the CP from the other hemisphere. The regression analysis explicitly discovered a positive correlation between the number of T cells and mRNA level of A_2A_R in the CP, indicating the functional linkage between abnormal A_2A_R signal in the CP and T cell trafficking (Fig. 1I). The parallel expression pattern and correlation analysis of the time window between A_2A_R expression and immune infiltration through the CP suggest that elevated A_2A_R signals (day 8–12) may facilitate T cell migration across the CP.

### Selective treatment with the A_2A_R antagonist KW6002 at post-immunization day 8–12 inhibits T cell infiltration into the CNS and EAE pathology

**Fig. 2:**
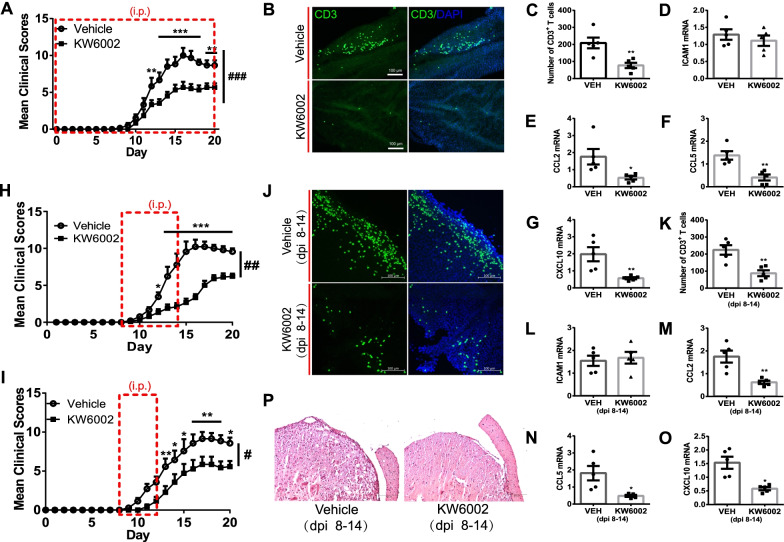
A_2A_R antagonist KW6002 attenuated EAE pathology in mice. **A** Full-phase injection (i.p.) of KW6002 decreased the clinical scores of EAE (*n* = 7/group). Two-way RM (repeated measures) ANOVA, Sidak’s multiple comparisons test. **B** Full-phase injection (i.p.) of KW6002 reduced the recruitment of CD3^+^ T cells on the CP (*n* = 5/group) at day 12 post-immunization. **C** Quantitative analysis of leukocytes CD3^+^ T cells on the CP in **B** (*n* = 5/group). Unpaired *t* test. **D**–**G** Full-phase injection (i.p.) of KW6002 downregulated the mRNA levels of *ccl2*, *ccl5* and *cxcl10* (*n* = 5/group). Unpaired *t* test. **H** Injection (i.p.) of KW6002 (day post-immunization (dpi) 8–14) reduced the clinical scores of EAE (*n* = 9/group). Two-way RM ANOVA, Sidak’s multiple comparisons test. **I** Injection (i.p.) of KW6002 (dpi 8–12) reduced the clinical scores of EAE (*n* = 7/group). Two-way RM ANOVA, Sidak’s multiple comparisons test. **J** Injection (i.p.) of KW6002 (dpi 8–14) reduced the recruitment of CD3^+^ T cells to the CP (*n* = 5/group) at day 12 after immunization. **K** Quantitative analysis of leukocytes CD3^+^ T cells on the CP in **J** (*n* = 5/group). Unpaired *t* test. **L**–**O** Injection (i.p.) of KW6002 (dpi 8–14) downregulated the mRNA levels of *ccl2*, *ccl5* and *cxcl10* (*n* = 5/group). Unpaired *t* test. **P** At dpi 14, the H&E staining of spinal cords from EAE mice injected (i.p.) with KW6002 or vehicle (dpi 8–14) (*n* = 5/group). ^#^*p* < 0.05, ^##^*p* < 0.01, ^###^*p* < 0.001, *p < 0.05, **p < 0.01 and ***p < 0.001

Given the selective upregulation of A_2A_R in parallel with the similar increase in leukocyte trafficking determinants and CD3^+^ T cell infiltration in the CP, we proposed that the A_2A_R antagonist may act on the CP to restrain the leukocyte infiltration across the CP and subsequently reduce the brain damage. Firstly, after the MOG_35-55_ immunization, mice were treated with an intraperitoneal injection (i.p.) of the A_2A_R antagonist KW6002 or vehicle every day for 20 days. Consistent with a previous study [13], KW6002 treatment protected against EAE pathology as evident from the reduced clinical scores (Fig. 2A). We then isolated the PBS-perfused CPs at day 14 and stained them with a CD3 antibody. Notably, large numbers of CD3^+^ T cells were detected in the CPs of the vehicle group, whereas few of CD3 positive signals were observed in the CPs of the KW6002-treated group (Fig. 2B, C). We also analyzed the leukocyte trafficking molecules (*icam1*, *ccl2*, *ccl5* and *cxcl10*) and found that the transcripts of *ccl2*, *ccl5* and *cxcl10* were sharply suppressed by KW6002, while the *icam1* level did not show any changes (Fig. 2D–G).

Based on the finding mentioned above that the phase of infiltration across the CP was at days 8–16, we immunized the mice with MOG_35-55_ and injected (i.p.) KW6002 or vehicle at days 8–14 or 8–12 (i.e., at the phase of infiltration phase). We found that time-phase administration (days 8–14 or 8–12) of KW6002 also effectively protected mice from EAE pathology compared to the vehicle group (Fig. 2H, I). This selective KW6002 treatment decreased T cell trafficking across the CP (Fig. 2J–O) and reduced the consequent mild immune infiltration in the spinal cord (Fig. 2P). This finding supported the idea that the CP is one of the critical action sites of the A_2A_R antagonist against EAE pathology.

### Tissue-specific knock-down of A_2A_R in the CP blunts its gateway activity and attenuates EAE pathology

**Fig. 3:**
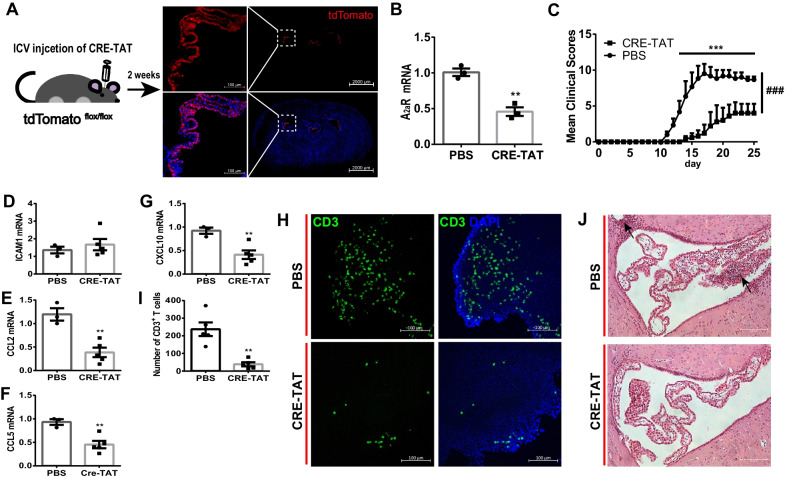
Knockdown of A_2A_R expression in the CP attenuated EAE pathology. **A** Representative image of tdTomato ^flox/flox^ mice treated with ICV-injection of CRE-TAT. Two weeks later, the expression of tdTomato was restricted to the CP (*n* = 3/group). **B** Two weeks after ICV-injection of CRE-TAT into A_2A_R ^flox/flox^ mice, the expression of A_2A_R in the CP was notably knocked down (*n* = 3/group). Unpaired *t* test. **C** Specific knock-down of A_2A_R in the CP reduces the clinical scores (*n* = 5–6/group). Two-way RM ANOVA, Sidak’s multiple comparisons test. **D-G** Specific knock-down of A_2A_R in the CP reduced the expressions of *ccl2*, *ccl5*, and *cxcl10* (*n* = 3/PBS group and *n* = 5/CRE-TAT group). Unpaired *t* test. **H** Specific knock-down of A_2A_R in the CP reduced the recruitment of CD3^+^ T cells to the CP at day 12 post-immunization (*n* = 3/group). **I** Quantitative analysis of leukocytes CD3^+^ T cells on the CP in **H** (*n* = 5/group). Unpaired *t* test. **J** The H&E staining showed that specific knock-down of A_2A_R in the CP attenuated the infiltration of immune cells in the CP at day 12 after immunization (*n* = 3/group). ^###^*p* < 0.001, *p < 0.05, **p < 0.01 and ***p < 0.001

Systemic administration of KW6002 at a specific time window (with a peak induction of A_2A_R) could not exclude the protective effects on sites other than the CP. Therefore, to isolate the CP as the key site for the A_2A_R-mediated protection in EAE pathology, we produced the local knock-down of A_2A_R in the CP by ICV-injection of CRE-TAT recombinase to A_2A_R^flox/flox^ transgenic line established in the lab. To confirm the CP-specificity of A_2A_R, we first injected the CRE-TAT protein into the lateral ventricles of mice expressing tdTomato fluorescent protein (RFP) under a loxP cassette. The analysis showed that the spontaneous red fluorescence was restricted to the ventricles and mainly located in the CP tissue (Fig. 3A).

Next, we performed ICV-injection of CRE-TAT into A_2A_R^flox/flox^ mice and then isolated the CP for determining the A_2A_R ablation in CP after 2 weeks. The qPCR analysis showed that A_2A_R transcripts were reduced by about 50%, compared to the PBS-treated group (Fig. 3B). Following EAE induction with MOG_35-55_ immunization, we found that the CP-A_2A_R knock-down mice just developed mild scores compared to the control group, consistent with the protective effect of KW6002 in the WT mice (Fig. 3C). Importantly, we found that CP-specific knock-down of A_2A_R prominently delayed the onset of EAE by 3 days, i.e., the first symptom detected at dpi (day post-immunization) 14 in the A_2A_R knock-down group (Fig. 3C).

We further assessed the contribution of A_2A_R signal in the CP to the gating of lymphocyte infiltration in a pathological lesion. The qPCR analysis also displayed that chemokine (*ccl2*, *ccl5,* and *cxcl10*) expression was decreased following down-regulation of A_2A_R signal in the CP (Fig. 3D–G). CD3 staining of the CP tissues showed that blocking A_2A_R in the CP could effectively decrease T cell recruitment in the CP (Fig. 3H, I). Using H&E staining, we observed dramatically reduced lymphocyte recruitment in the CP tissue and infiltration in the brain parenchyma, as well as ameliorated BBB leakage with no “perivascular cuffs” in the A_2A_R-knock-down group (Fig. 3J). Overall, these results indicated that blocking A_2A_R signal in the CP was sufficient to control the EAE pathological process.

### A_2A_R signal in the CP prevents Th17^+^ T cells from infiltrating into the CSF via inhibiting the CCR6–CCL20 axis

**Fig. 4:**
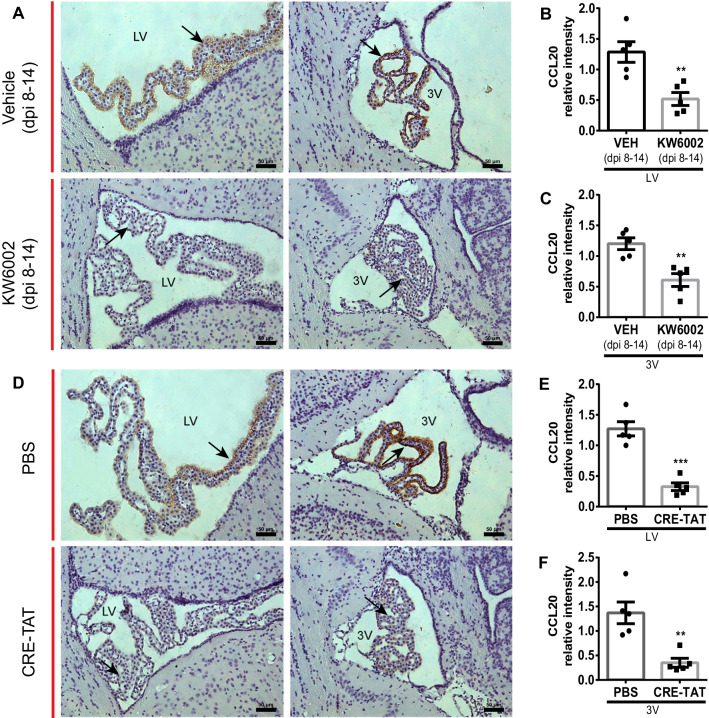
Knockdown of A_2A_R expression in the CP reduced the expression of CCL20. **A** Injection (i.p.) of KW6002 (dpi 8–14) reduced the expression of CCL20 on the CP at day 14 after immunization (*n* = 5/group). **B**, **C** Quantitative analysis of CCL20 in the CP in **A** (*n* = 5/group). Unpaired *t* test. **D** specific knock-down of A_2A_R in the CP inhibited the expression of CCL20 in CP at day 14 after immunization (*n* = 5/group). **E–F** Quantitative analysis of CCL20 in the CP in **D** (*n* = 5/group). Unpaired *t* test. **p < 0.01 and ***p < 0.001. “LV” = the lateral ventricle, and “3V” = the third ventricle

During EAE induction, the CCR6–CCL20 axis was necessary for the first wave of Th17^+^ T cells, which entered the CNS through the CP epithelium in the EAE pathology [26]. Furthermore, CCL20 was enriched on the CP epithelium under healthy or EAE pathological conditions [5]. We next investigated whether KW6002 or CP-specific knock-down of A_2A_R inhibited the rolling of Th17^+^ cells into the CNS across the CP by disturbing the CCR6–CCL20 axis. The immunohistochemical analysis showed that CCL20 was highly expressed in the CP at day 14 after EAE induction (Fig. 4A–F). Significantly, KW6002 administration (i.p., day 8–14) and the CP-specific knock-down of A_2A_R largely abolished CCL20 upregulation in the CP (Fig. 4B, C and E, F).

**Fig. 5:**
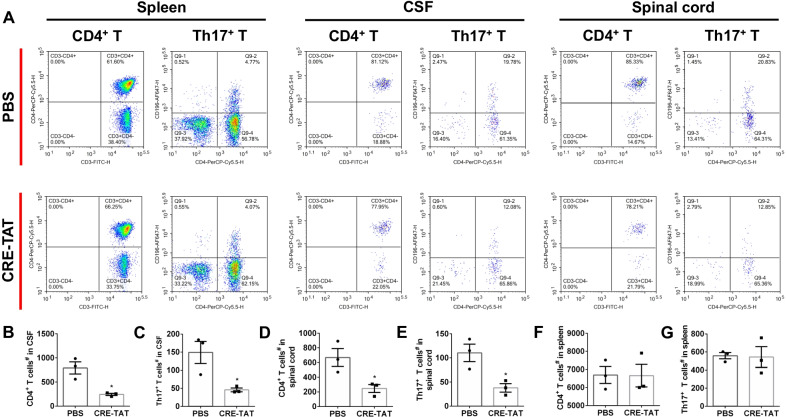
Knockdown of A_2A_R expression in the CP inhibited the infiltration of Th17^+^ T cells. **A** Following knock-down of A_2A_R signal in the CP and EAE induction, flow cytometry was performed to analyze the number of CD4^+^ or Th17^+^ T cells in the CSF, spinal cords, and spleen at day 14 post-immunization. **B**, **C** Knockdown of A_2A_R expression in the CP reduced the infiltration of CD4^+^ or Th17^+^ T cells in the CSF (*n* = 3/group) at day 14 post-immunization. Unpaired *t* test. **D**, **E** Knockdown of A_2A_R expression in the CP reduced the infiltration of CD4^+^ or Th17^+^ T cells in the spinal cord (*n* = 3/group) at day 14 post-immunization. Unpaired *t* test. **F**, **G** Knockdown of A_2A_R expression in the CP did not alter the number of CD4^+^ or Th17^+^ T cells in the spleen (*n* = 3/group) at day 14 post-immunization. Unpaired t test. *p < 0.05

We further determined the effect of CP-A_2A_R knock-down on Th17^+^ infiltration across the CP. We first collected and analyzed the changes of CD4^+^ T cells in the CSF by flow cytometry on the 14^th^ day after EAE induction (Fig. 5A, Additional file 3: Fig. S3). As expected, large numbers of CD4^+^ T cells, existed in the CSF of the WT mice that developed EAE. However, only a handful of CD4^+^ T cells was detected in the CSF of mice treated with TAT-CRE (Fig. 5A, B). Moreover, we evaluated the effect of the CP-specific knock-down of A_2A_R on Th17^+^ T cells (a pathogenic CD4^+^ T cell subpopulation). Similarly, blocking A_2A_R in the CP largely abolished infiltration of the Th17^+^ T cells in the CSF (Fig. 5C). Next, we investigated the effect of the CP-specific knock-down of A_2A_R on the population of Th17^+^ T cells in the spinal cord. Like the CSF effect, the number of Th17^+^ T cells was reduced in the CRE-TAT group compared to the control group (Fig. 5D, E). By contrast, the CP-specific knock-down of A_2A_R did not alter the amount of Th17^+^ T cells in the spleen (Fig. 5F, G). Collectively, these findings supported the idea that A_2A_R signal in the CP prevented Th17^+^ T cells from infiltrating into the CSF and the brain via the CCR6–CCL20 axis.

### Enhanced A_2A_R signaling in the CP epithelium induces chemokine activity and disrupts the tight junctions

**Fig. 6:**
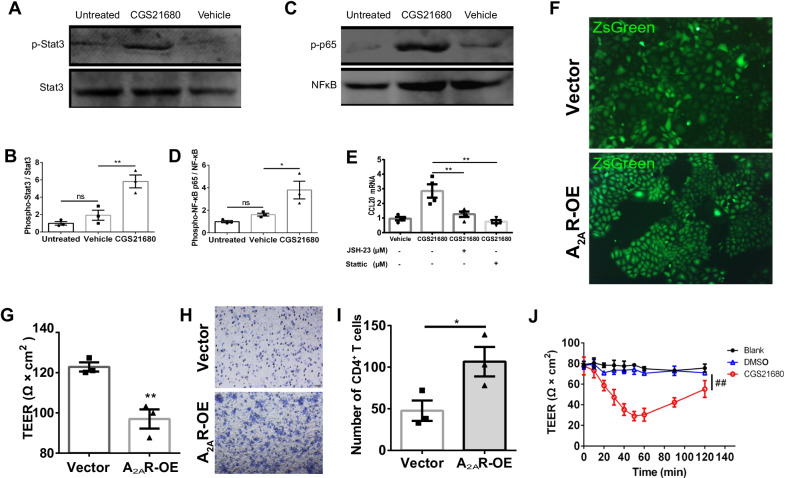
Elevation of A_2A_R signal in the CP epithelium increased the permeability and promoted the migration of T cells. **A**, **B** In the primary CP epithelium, the A_2A_R agonist CGS21680 (100 nM, treatment for 2 h) increased the phosphorylation level of STAT3 (*n* = 3/group). **C**, **D** In the primary CP epithelium, the A_2A_R agonist CGS21680 (100 nM, treatment for 2 h) also increased the phosphorylation level of p65 (*n* = 3/group).** E** Pre-treatment with NF-κB transcriptional activity inhibitor (JSH-23, 10 μM) or specific STAT3 inhibitor (Stattic, 100 μM) for 1 h abolished the increased expression of *ccl20* induced by CGS21680 (100 nM, treatment for 2 h) in the primary CP epithelium (*n* = 4/group). **F** Representative image of ZsGreen-labeled A_2A_Rs overexpressed in the Z310 cell line. **G** Over-expression of A_2A_R increased the resistance value of Z310 cells (*n* = 3/group). Unpaired *t* test. **H**, **I** Over-expression of A_2A_R promoted the migration of CD4^+^ T cells across the Z310 cell layer (*n* = 3/group). Unpaired *t* test. **J** Following the addition of CGS21680 into the medium, the resistance values of the primary CP epithelium were monitored in real-time within 2 h (*n* = 3/group). One-way RM ANOVA, Dunnett’s multiple comparisons test. ^##^*p* < 0.01, *p < 0.05 and **p < 0.01. “p” stands for phosphorylation

To find out whether A_2A_R acts at the CP epithelium (from multiple cell types in the CP) to confer protection, we investigated the effect of A_2A_R activity on chemokine signaling and tight junctions in the primary CP epithelium. We first probed the NFκB/STAT3 signaling mechanism of A_2A_R regulating CCL20 by stimulating the A_2A_R signal in the CP epithelium with the A_2A_R agonist CGS21680. The western blot analysis revealed that CGS21680 treatment increased the level of phosphorylated p65 (Ser536) and STAT3 (Tyr705) compared to the vehicle group (Fig. 6A–D, additional file 4: Fig. S4). In parallel with p65 (Ser536) and STAT3 (Tyr705), the level of *ccl20* mRNA was also induced by CGS21680 (Fig. 6E). Furthermore, we combined the inhibitor of NFκB or STAT3 pathway with CGS21680 and found that the upregulation of *ccl20* induced by CGS21680 was abolished (Fig. 6E).

Next, we determined the effect of elevated A_2A_R signal on the TJ function and leukocyte migration across the CP as TJ structure formed by the CP monolayer epithelium was the gateway to prevent the entry of macromolecules. We firstly overexpressed *Adora2a* (mouse, NM_009630.3) in the CP epithelium cell line (rat, Z310) (Fig. 6F). We found that the overexpression of A_2A_R significantly decreased the values of electrical resistance, which indicated that activation of A_2A_R increased the permeability of TJ (Fig. 6G). Moreover, the trans-epithelial migration assay revealed that increased A_2A_R signal facilitated the migration of T lymphocytes across epithelial barriers (Fig. 6H, I). In addition, after culturing the murine primary CP epithelium for 5 days, the A_2A_R agonist CGS21680 was added into the chambers. The dynamic curves showed that the permeability of the epithelium was rapidly increased within 1 h (Fig. 6J). This suggested that elevated A_2A_R in the CP could compromise TJ integrity and facilitate lymphocyte trafficking in EAE.

## Discussion

Identification and characterization of the effective molecular targets in the CP to control the heightened influx of immune cells across the CP are essential to elucidate the initial mechanisms driving MS pathogenesis and may lead to a novel pharmacological strategy for controlling autoimmune damage to CNS tissues. Our finding identifies A_2A_R signaling in the CP niche as a key controller for T cell trafficking and EAE pathology. (1) Following the MOG_35-55_ immunization, phase (i.e., day 8–14 post-immunization) of elevated A_2A_R expression coincided with the early T cell infiltration across the CP and attenuated behavioral deficit in the EAE mice. (2) Consistent with the peak A_2A_R expression window, the A_2A_R antagonist KW6002 (administered at post-immunization day 8–12 or 8–14) markedly reduced the EAE-induced infiltration of CD3^+^ T cells across the CP and attenuated behavioral deficit in the EAE mice. (3) By targeted deletion of A_2A_R in the CP via local injection of CRE-TAT recombinant protein into the A_2A_R^flox/flox^ mice, we found that the CP-specific knock-down of A_2A_R effectively reduced the infiltration of Th17^+^ cells in the CSF and spinal cord and delayed the onset of EAE behavioral deficit and ameliorated the scores. This was accomplished by CP-A_2A_R modulation of the expression of *ccl20* in the CP epithelium. (4) A_2A_R control of the CP gateway activity is at least partially mediated by direct action on the CP epithelium since activation of A_2A_R signal (by overexpression or agonist) in the CP epithelium increased the permeability and facilitated lymphocytes migration, in agreement with the previous study showing that the A_2A_R agonist promotes lymphocyte transmigration across CPLacZ-2 cells [19]. (5) Blocking A_2A_R signal in the CP can prevent Th17^+^ T cells from infiltrating into the CSF by inhibiting the CCR6–CCL20 axis and NFκB/STAT3 pathway. Collectively, these findings defined (abnormally elevated) A_2A_R signaling in the CP niche as a critical modulator for the CP gateway activity whereby lymphocytes infiltrated into the CNS via the BCSFB route during the first wave of T cell infiltration in EAE development. These findings further solidify the essential role of the CP gateway activity in EAE development, particularly the first wave of T cell infiltration.

The identification of the CP A_2A_R activity controlling the CP gateway and EAE pathology also provides a partial resolution for the paradoxical (opposite) effects of A_2A_R knockout and A_2A_R antagonist on EAE pathology, Moreover, this finding identified A_2A_R in the CP as the source of non-hematopoietic cells that confer the A_2A_R-mediated protection as revealed by the adoptive transfer experiment [17]. While the protective effect of the A_2A_R antagonists (SCH58261 and caffeine) has been well demonstrated in the EAE model [13, 14], the paradoxical exacerbation of EAE pathology by A_2A_R KO complicated the therapeutic development of selectively targeting A_2A_R [16]. Notably, A_2A_R antagonists protect against EAE pathology even in the presence of pro-inflammatory A_2A_R^−/−^ CD4^+^ T cells as administering the A_2A_R antagonist SCH58261 protected against EAE pathology following the adoptive transfer of WT or A_2A_R^−/−^ CD4^+^ T cells into the A_2A_R^+/+^ TCR-deficient mice [17]. Our finding that targeted deletion of A_2A_R in the CP protects mice from EAE induction provides a direct evidence that the protective effects of A_2A_R antagonists on non-hematopoietic cells are likely mediated by A_2A_R signaling in the CP, which modulates the lymphocyte migration into the CNS.

Critically, we demonstrated that the timing of A_2A_R administration (post-immunization day 8–12) is critical for the successful treatment of MS by A_2A_R antagonists. The leukocyte trafficking across the CP was considered to be the first wave of T cell trafficking in EAE development (not an initiation of autoimmune response). A_2A_R signaling has been shown to play a distinct role at the different stages of EAE developmental course. Specifically, during the initiation stage, (post-MOG immunization day 0–10), administering an A_2A_R agonist on dpi 0–16 conferred protection while SCH58261 administration on day 0–10 displayed no protective effect [13, 23]. Adoptive transfer experiments demonstrated that the mechanism of such protection (A_2A_R agonist effect) to be the downregulated inflammatory potential of A_2A_R-expressing lymphocytes [23]. During EAE disease progression, administering A_2A_R antagonist on day 11–28 (peak of symptomatic disease) conferred protection while administering the A_2A_R agonist had a detrimental effect [13, 23]. However, this disease progression stage is characterized by two steps in disease development: the first wave of encephalitogenic Th17 cells infiltration into the CNS via the CP epithelium in a CCL20-dependent gradient, and the subsequent recruitment of both encephalitogenic T cells and bystander cells, such as macrophages occurs via the BBB (postcapillary venules) within the brain parenchyma. Importantly, two lines of our experimental findings further narrow the effective therapeutic window of KW6002 to the day 8–12 for protection against EAE pathology: (1) there was a specific peak induction of A_2A_R expression in the CP around dpi 12, in agreement with the upregulation of CD73 [18] and CGS21680-regulated CX3CL1 expression [19] at this time window; (2) administration of KW6002 selectively during the acute stage (i.e., day 8–12) can alleviative pathology with the reduced immune infiltration across the CP. Thus, systemic administration of KW6002 at the specific acute stage in EAE (dpi 8–12), namely the time window with the intense T cell infiltration across the CP, may effectively confer protection as offered by KW6002 treatment throughout the entire disease progression course. This strongly argues that the CP niche is one of the critical sites where KW6002 controls the first wave of blood lymphocytes migration into the CSF, which may, in turn, weaken the BBB destruction by pro-inflammatory factors from the CSF. This also suggests that systemic administration of KW6002 at this specific time window (with enhanced CP A_2A_R signaling) may be sufficient to accomplish selective targeting of the CP-A_2A_R.

Identifying the ability of the CP A_2A_R signaling to control the CP gateway for T cell trafficking confers A_2A_R antagonists an ability to protect against EAE pathology by directly targeting the CP epithelium (to control immune cell trafficking, rather than immune cell activity directly). The clinical studies have demonstrated the clinical effectiveness of the natalizumab to control immune infiltration for clinical MS treatment by blocking the binding between VLA-4 and VCAM [2]. Notably, all these FDA-approved MS drugs are designed to directly target immune cells, which may completely inhibit lymphocyte migration. Such complete inhibition may be associated with the consequential induction of temporary immunodeficiency in patients, such as the increased risk of progressive multifocal leukoencephalopathy (PML) and other opportunistic infections [27]. Blockade of A_2A_R signaling reduced the expression of *ccl20* and inhibited the CCR6–CCL20 axis in the CP via the NFκB/STAT3 signaling cascade. This indirect modulation of chemotaxis (CCR6–CCL20) and adhesion molecules in the choroid plexus may offer a better approach to treating MS by selectively interfere with the recruitment of pathogenic leukocytes to the CNS while leaving host protective immune mechanisms intact (i.e., by inhibiting certain, but not all, lymphocyte subsets from entering the CNS). Thus, the identification of the CP A_2A_R activity to control the CP epithelium for T cell trafficking and EAE pathology suggests a novel strategy for alleviating EAE pathology solely by targeting the CP epithelium activity, rather than directly targeting immune cell activity, to avoid the unwanted side effects. The recent approval of the A_2A_R antagonist Nourianz^®^ (istradefylline) by the U.S. Food and Drug Administration (FDA) as an add-on treatment to levodopa in Parkinson’s disease (PD) with “OFF” episodes in 2019 [28] have demonstrated its noted safety profile and clinical utility. This approval offers new therapeutic opportunities for translating the A_2A_R antagonist for the disease-modifying treatment of MS.

## Conclusion

This study provides the direct evidence in establishing a critical role of the CP-A_2A_R in control of T cell infiltration and EAE pathology: (1) A_2A_R signal was upregulated in the CP tissues at day 8–12 post-immunization, correlating with the increased CP gateway activity and T cell infiltration across the CP; (2) the selective treatment with the A_2A_R antagonist KW6002 at post-immunization days 8–12 or 8–14 was sufficient to reduce T cell trafficking across the CP and attenuate EAE pathology; (3) selective knock-down of A_2A_Rs in the CP attenuated Th17 cells infiltration and alleviated EAE pathology; (4) CP-A_2A_R controlled T cell trafficking by inhibiting the CCR6–CCL20 axis, partially by direct A_2A_R action on the CP epithelium. Collectively, these findings define the CP niche as one of the primary sites for the A_2A_R antagonist to confer protection against EAE pathology. Thus, pharmacological targeting of the CP-A_2A_R represents a novel treatment strategy for MS by controlling immune cell trafficking across the CP.

## Supplementary Information

**Additional file 1: Figure S1.** The mRNA expression of A_2A_R in striatum (Str), hippocampus (Hip), cortex (Cx) and choroid plexus (CP) of wild type mice (*n* = 4/group).

**Additional file 2: Figure S2.** Immune infiltration into CNS through the CP happened ahead of the BBB in EAE model. **A** Following EAE induction in WT mice, the HE staining was performed at day 0, 4, 8, 12 and 16. Scale bar, 100 µm (*n* = 3/group). **B** Representative dextran-stained brains from WT mice at day 8, 10 and 12 following EAE induction. Scale bar, 100 µm (*n* = 3/group).

**Additional file 3: Figure S3.** Gating strategy and representative flow cytometry plots of FVS510, CD3-FITC, CD4-PerCP and CD196-AF647 in the CSF, spinal cord and spleen (*n* = 3/group). Dead cells: FVS510^+^, live cells: FVS510^low^, CD4^+^ T cells: FVS510^low^ CD3^+^ CD4^+^, Th17^+^ T cells: FVS510^low^ CD3^+^ CD4^+^ CD196^+^.

**Additional file 4: Figure S4.** The original images of Western Blot. **A** The WB images of p-p65 and p65. **B** The WB images of p-STAT3 and STAT3. p was phosphorylation. The lanes of 1, 4 and 7 belong to the group of untreated primary CP epithelium, lanes of 2, 5 and 8 belong to the group of primary CP epithelium treated with CGS21680, and lanes of 3, 6 and 9 belong to the group of primary CP epithelium treated with vehicle.

## Data Availability

The data used in this study are available from the corresponding author on reasonable request.

## Abbreviations

MS: Multiple sclerosis
CP: Choroid plexus
EAE: Experimental autoimmune encephalomyelitis
CNS: Central nervous system
A_2A_R: Adenosine A2A receptor
MOG: Myelin oligodendrocyte glycoprotein
BBB: Blood–brain barrier
TJ: Tight junction
CSF: Cerebrospinal fluid
PET: Positron emission tomography
SPMS: Secondary progressive multiple sclerosis
CPE: Choroid plexus epithelium
FISH: Fluorescence in situ hybridization
NECA: Non-specific adenosine receptor agonist
MOI: Multiplicity of infection
FBS: Fetal bovine serum
Ara-C: Arabinosylcytosine
EGF: Epidermal growth factor
RM: Repeated-measurements
ICAM-1: Intercellular cell adhesion molecule-1
LFA-1: Lymphocyte function-associated antigen-1
BCSFB: Blood–CSF barrier
PML: Progressive multifocal leukoencephalopathy
FDA: Food and Drug Administration
PD: Parkinson’s disease
Str: Striatum
Hip: Hippocampus
Cx: Cortex
i.p.: Intraperitoneal injection
dpi: Day post-immunization
VEH: Vehicle
