# Supplementary figures and images for: Choroid plexus-selective inactivation of adenosine A2A receptors protects against T cell infiltration and experimental autoimmune encephalomyelitis
Source: J Neuroinflammation. 2022 Feb 18;19:52. doi: 10.1186/s12974-022-02415-z (PMC8855604; doi:10.1186/s12974-022-02415-z)

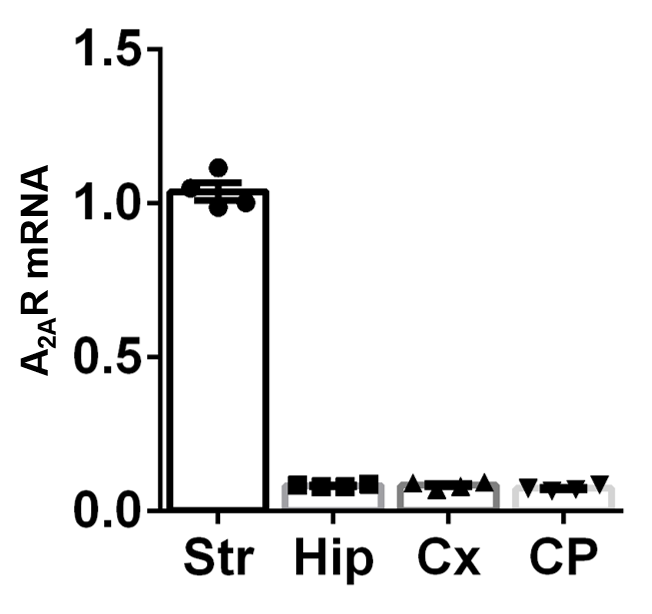

Supplement: Supplementary file 1 — Additional file 1: Figure S1. The mRNA expression of A2AR in striatum (Str), hippocampus (Hip), cortex (Cx) and choroid plexus (CP) of wild type mice (n = 4/group). [file 12974_2022_2415_MOESM1_ESM.tif]

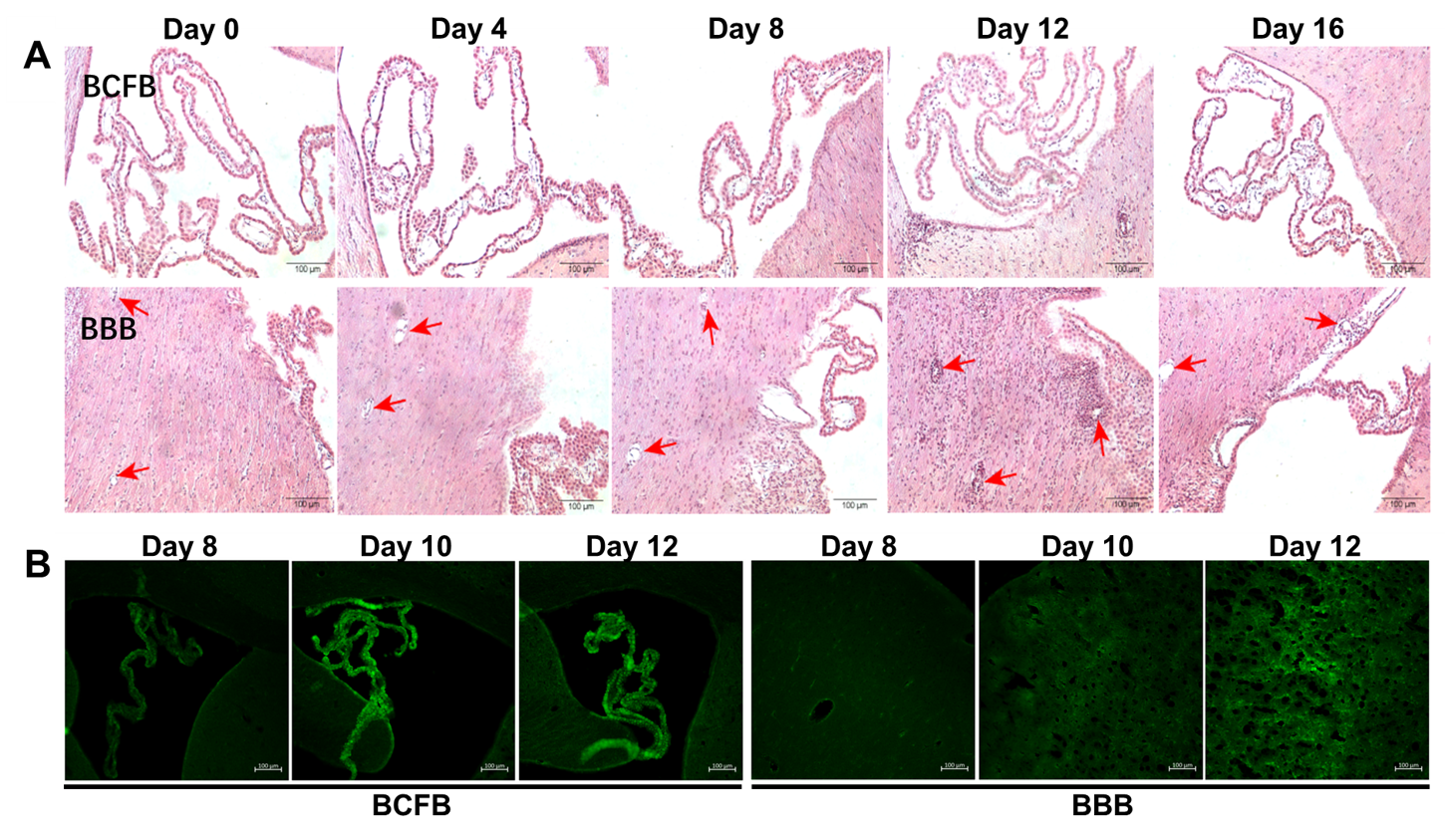

Supplement: Supplementary file 2 — Additional file 2: Figure S2. Immune infiltration into CNS through the CP happened ahead of the BBB in EAE model. A Following EAE induction in WT mice, the HE staining was performed at day 0, 4, 8, 12 and 16. Scale bar, 100 µm (n = 3/group). B Representative dextran-stained brains from WT mice at day 8, 10 and 12 following EAE induction. Scale bar, 100 µm (n = 3/group). [file 12974_2022_2415_MOESM2_ESM.tif]

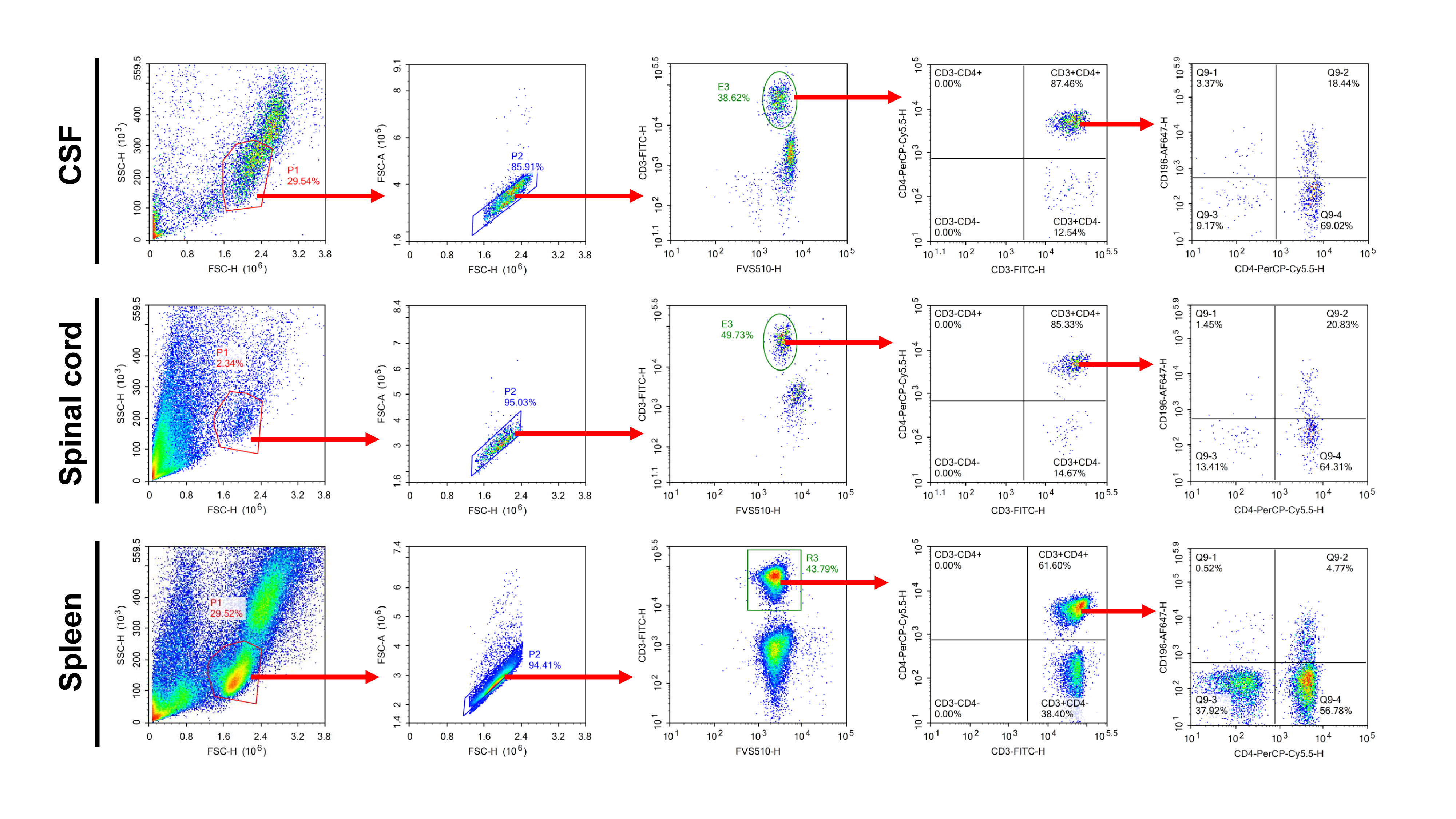

Supplement: Supplementary file 3 — Additional file 3: Figure S3. Gating strategy and representative flow cytometry plots of FVS510, CD3-FITC, CD4-PerCP and CD196-AF647 in the CSF, spinal cord and spleen (n = 3/group). Dead cells: FVS510+, live cells: FVS510low, CD4+ T cells: FVS510low CD3+ CD4+, Th17+ T cells: FVS510low CD3+ CD4+ CD196+. [file 12974_2022_2415_MOESM3_ESM.tif]

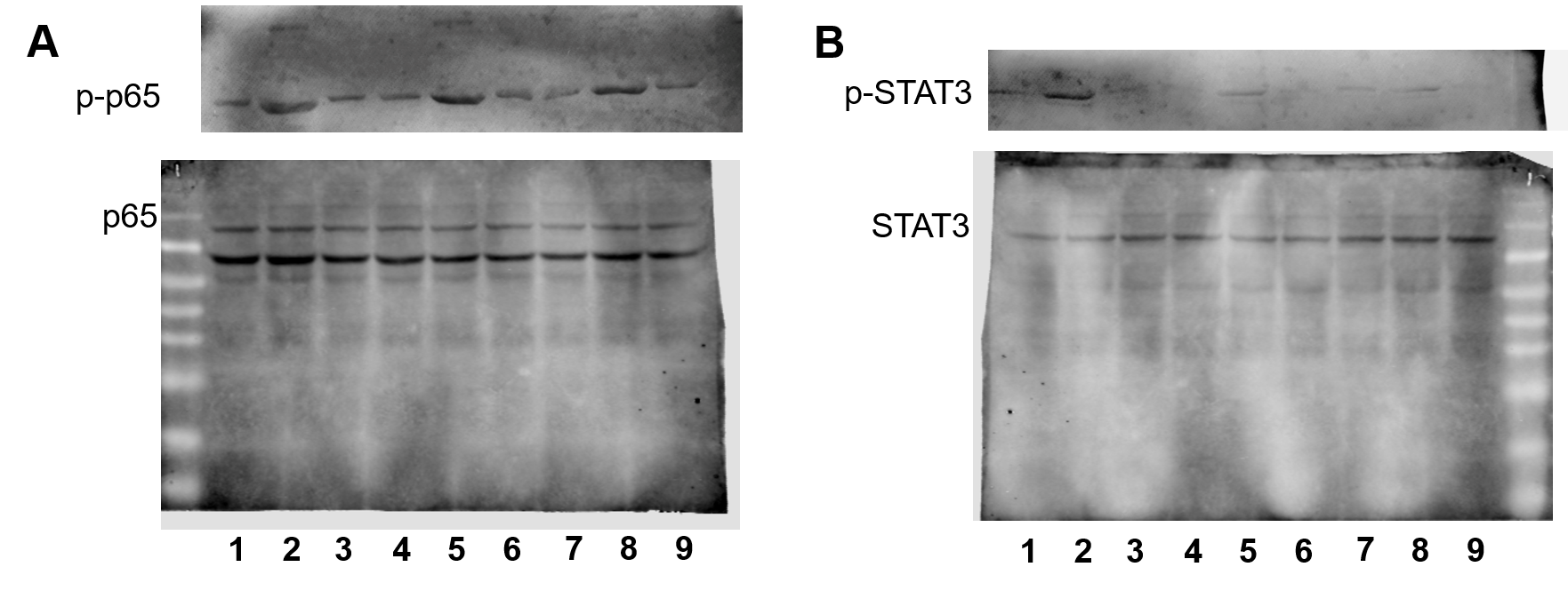

Supplement: Supplementary file 4 — Additional file 4: Figure S4. The original images of Western Blot. A The WB images of p-p65 and p65. B The WB images of p-STAT3 and STAT3. p was phosphorylation. The lanes of 1, 4 and 7 belong to the group of untreated primary CP epithelium, lanes of 2, 5 and 8 belong to the group of primary CP epithelium treated with CGS21680, and lanes of 3, 6 and 9 belong to the group of primary CP epithelium treated with vehicle. [file 12974_2022_2415_MOESM4_ESM.tif]
